# Multiplicity of benign breast disease lesions and breast cancer risk in African American women

**DOI:** 10.3389/fonc.2024.1410819

**Published:** 2024-05-16

**Authors:** Vidya Patil, Julie J. Ruterbusch, Wei Chen, Julie L. Boerner, Eman Abdulfatah, Baraa Alosh, Visakha Pardeshi, Asra N. Shaik, Sudeshna Bandyopadhyay, Rouba Ali-Fehmi, Michele L. Cote

**Affiliations:** ^1^ Simon Comprehensive Cancer Center, Indiana University, Indianapolis, IN, United States; ^2^ Department of Pathology, Wayne State University School of Medicine, Detroit, MI, United States; ^3^ Department of Oncology, Wayne State University School of Medicine, Detroit, MI, United States

**Keywords:** African American, breast cancer, benign breast disease, breast biopsy, hyperplasia

## Abstract

The risk of developing subsequent breast cancer is higher in women diagnosed with benign breast disease (BBD) but these studies were primarily performed in non-Hispanic white populations. Still, these estimates have been used to inform breast cancer risk models that are being used clinically across all racial and ethnic groups. Given the high breast cancer mortality rates among African American (AA) women, it is critical to study BBD in this population, to ensure the risk models that include this information perform adequately. This study utilized data from AA women who underwent benign breast biopsies at a hospital served by the University Pathology Group in Detroit, Michigan, from 1998 to 2010. Patients were followed for subsequent breast cancers through the population-based Metropolitan Detroit Cancer Surveillance System (MDCSS). BBD lesion scores were assigned to represent the severity or extent of benign breast lesions, with higher scores indicating a greater number of distinct lesion types. Of 3,461 eligible AA women with BBD in the cohort, 6.88% (n=238) subsequently developed breast cancer. Examined individually, six of the eleven lesions (apocrine metaplasia, ductal hyperplasia, lobular hyperplasia, intraductal papilloma, sclerosing adenosis, columnar alterations and radial scars) were significantly associated with increased risk of breast cancer after adjustment for age and year of biopsy and were further considered in multiple lesion models. For every different type of benign breast lesion, subsequent risk of breast cancer increased by 25% (RR=1.25, 95% CI: 1.10, 1.42) after adjustment for age at biopsy and proliferative versus non-proliferative disease. In summary, this study affirms the increased breast cancer risk in AA women with BBD, particularly in those with multiple lesions. These findings have implications for the management of breast cancer risk in millions of women affected by BBD, a high risk group that could benefit from personalized surveillance and risk reduction strategies.

## Introduction

In the United States, African American (AA) women have the highest breast cancer (BC) mortality rates compared to other racial and ethnic groups. Additionally, the incidence rates among AA women are also higher than all other racial and ethnic groups, other than non-Hispanic white women (1). First described by Dupont et al. in 1980, women diagnosed with benign breast disease (BBD) have a higher risk of subsequent development of BC (2). Based on this seminal work, BBD lesions are often categorized into three groups: non-proliferative disease (NPD), proliferative disease without atypia (PDWA), and atypical hyperplasia (AH). Of these, AH is associated with the greatest subsequent risk of BC, with approximately 1 in 4 women developing a subsequent BC over the next two decades (3). AH has been identified in up to 10% of BBD biopsies, and randomized, controlled trials with existing cancer prevention therapies have shown a substantial risk reduction benefit (4). PDWA, representing ~40% of all BBD, is associated with at least a 2-fold increase in BC risk. Even biopsies that show NPD, the lesions with lowest risk, were still associated with risk that is 25% greater than that of women who have never undergone a clinical biopsy. As reviewed by Dyrstad et al, women with proliferative types of benign breast disease, both with and without atypia, have increased risk of BC (5).

Despite several decades of research, the studies that have examined BBD have been primarily comprised of non-Hispanic white women (3, 6–8). Given the earlier onset of disease and the poorer prognosis experienced by AA women, it is critical to better define the risk of BBD lesions and subsequent risk of BC in this population. Here, we assess the association between various types of BBD lesions and subsequent BC, quantify the BC risk associated with multiplicity of these lesions, and provide a description of the types of BC that have developed in this high-risk cohort of AA women with BBD.

## Methods

### Study population

The Detroit Benign Breast Disease Cohort (BBD Cohort) is comprised of n=3,860 AA women (self-reported race obtained via medical record abstraction) who had a benign breast biopsy at a hospital served by the University Pathology Group from 1997-2010. Patients were followed for subsequent BCs, defined as an invasive or *in situ* cancer that occurred at least 6 months after the date of the benign biopsy, through the Metropolitan Detroit Cancer Surveillance System (MDCSS), a founding site of the National Cancer Institute’s Surveillance, Epidemiology and End Results (SEER) program. The last linkage to MDCSS was May 19, 2022; so, for participants who did not have diagnosis of BC or death, this was the date of censor for all analyses. Cote et al. provided more details regarding the study design (9). This study was approved by the Wayne State University Institutional Review Board (#087812M1E).

All the hematoxylin and eosin (H & E)-stained benign biopsy slides from each case (ranging from 2-22 slides per case) were retrieved from the Department of Pathology at Wayne State University and assessed for 11 different benign lesions by the study pathologist (RAF): apocrine metaplasia, ductal hyperplasia, lobular hyperplasia, calcifications, cysts, duct ectasia, fibrosis, intra-ductal papilloma, sclerosing adenosis, columnar alterations, and radial scar. Using these markers, the study pathologist also categorized the case using the criteria established by Dupont and Page: non-proliferative disease, proliferative disease without atypia, and proliferative disease with atypia (atypical hyperplasia). For the purposes of this analysis, women with atypical hyperplasia were removed from the study population (n=149), as there is a known, strong association with risk of subsequent BC (3). In addition, 250 cases were excluded that had slides containing only a large fibroadenoma where additional BBD features could not be assessed. Therefore, the dataset used in this analysis included a total of 3,461 participants.

### Laboratory analysis

To confirm the BC subtype listed in the original diagnostic pathology reports, we utilized formalin fixed, paraffin-embedded tumor blocks from a subset of women (n=100) identified with a subsequent cancer to examine the following markers: estrogen receptor (ER), progesterone receptor (PR), and human epidermal growth factor receptor 2 (HER2). Briefly, the slides were deparaffinized and rehydrated. Antigen retrieval was performed, followed by immunohistochemical staining using a Ventana automated immunohistochemical stainer, counterstained (when necessary), dehydrated, and mounted. The pathologist was blinded to the clinical characteristics and prior pathology report associated with the tissue. All antibodies were sourced from DAKO, and the specific conditions and positivity assessment are as follows: ER, clone 1D5, 1:100 dilution, positive if greater than 1% of nuclei stained; PR, clone PgR636, 1:100 dilution, positive if greater than 1% of nuclei stained; HER2, Hercept Test (pre-diluted), greater than 30% of cells showed circumferential intense and uniform staining. There was approximately a 90% concordance between the prior reports and the repeated analysis (data not shown), thus the receptor data abstracted from the pathology reports was used.

### Statistical analysis

This was a retrospective cohort study. Cases were classified as women who had a subsequent BC at least six months after their initial biopsy. Follow up time in months was calculated from date of biopsy to the date of BC diagnosis or death, whichever occurred first. Women were classified as controls if there was no record of a subsequent BC in MDCSS, and their follow up time was calculated from date of biopsy to: 1) death from other cause or 2) date of last cohort-MDCSS linkage.

Baseline characteristics were compared between cases and controls with chi-square tests for categorical variables and Wilcoxon’s rank sum test for continuous variables. The relative risk (RR) for each marker was estimated with logistic regression, adjusted for age and year of biopsy. A score was created by summarizing the number of markers which had univariate logistic regression p<0.05: ductal hyperplasia, lobular hyperplasia, intra-ductal papilloma, sclerosing adenosis, columnar alterations, and radial scars. The score ranged from 0 to 6, with 0 representing “no lesions” and 6 meaning that all 6 distinct types of lesions were present. All statistical tests were two sided, but p- values should be interpreted with caution due to issues of multiplicity testing. The widths of the 95% confidence intervals were not adjusted for multiplicity testing and cannot be used in place of a hypothesis test. All statistical analyses were performed using SAS software version 9.4 (Cary, NC).

## Results

Among the 3,461 eligible AA women with BBD in our study, 238 women subsequently developed BC. As shown in **Table 1**, those who developed BC were slightly older than those who did not (51 years versus 47 years at initial benign biopsy) and were more likely to have proliferative disease without atypia compared to non-proliferative lesions (p=0.001).

**Table 1:** Characteristics of women with benign breast disease by case/control status in the Detroit BBD Cohort.

Variable	Control (n=3,223)	Case (n=238)	p value
Age at Biopsy– median years (range)	47 (18,84)	51 (26,81)	**<0.001**
Follow up – median years (range)	16.9 (0.5,23.0)	8.9 (0.7,23.4)	**<0.001**
Biopsy year - no. (%)			0.473
1997-2000	1,131 (35)	89 (37)	
2001-2010	2,092 (65)	149 (63)	
Dupont and Page Classification - no. (%)			**0.001**
Non-Proliferative Disease	1,889 (59)	114 (48)	
Proliferative Disease without Atypia	1,334 (41)	124 (52)	

Bold values indicate statistical significance at the p < 0.05 level.

The distribution of 11 different types of benign breast diseases is shown in **Table 2**, along with the risk of developing BC associated with each type of lesion, adjusted for age and the year of the initial biopsy. There were six lesions that were associated with increased risk of subsequent BC that were used to create the score variable described in **Table 3**: ductal hyperplasia, lobular hyperplasia, intra-ductal papilloma, sclerosing adenosis, columnar alterations and radial scars. Overall, pathologic classification of proliferative disease without atypia was associated with a 57% increase in risk of developing a subsequent cancer compared to those biopsies classified as non-proliferative, but this composite variable was not included in the score variable classification.

**Table 2:** Distribution of type of benign lesion and risk of subsequent breast cancer in the Detroit BBD Cohort.

Variable	Control		Case			
	N	%	N	%	RR*	(95% CI)
**Total**	3,223		238			
**Apocrine Metaplasia**					1.06	0.80-1.40
No	2,250	70%	159	67%		
Yes	973	30%	79	33%		
**Ductal Hyperplasia**					**1.39**	**1.05-1.84**
None	2,345	73%	153	64%		
Yes	878	27%	85	36%		
**Lobular Hyperplasia**					**10.96**	**2.90-41.41**
None	3,218	100%	234	98%		
Yes	5	0%	4	2%		
**Calcifications**					1.04	0.78-1.39
No	2,109	65%	139	58%		
Yes	1,114	35%	99	42%		
**Cyst**					1.19	0.91-1.55
No	2,005	62%	135	57%		
Yes	1,218	38%	103	43%		
**Duct Ectasia**					1.14	0.79-1.64
No	2,753	85%	201	84%		
Yes	470	15%	37	16%		
**Fibrosis**					1.13	0.86-1.48
No	1,485	46%	100	42%		
Yes	1,738	54%	138	58%		
**Intra-Ductal Papilloma**					**1.61**	**1.13-2.28**
None	2,861	89%	195	82%		
1 or more	362	11%	43	18%		
**Sclerosing adenosis**					**1.80**	**1.34-2.41**
No	2,450	76%	160	67%		
Yes	773	24%	78	33%		
**Columnar alterations**					**1.86**	**1.41-2.46**
No	2,436	76%	148	62%		
Yes	787	24%	90	38%		
**Radial Scar**					**2.13**	**1.11-4.12**
No	3,157	98%	227	95%		
Yes	66	2%	11	5%		
Proliferative Disease						
No	1,889	59%	114	48%	**1.57**	**1.20-2.05**
Yes	1,334	41%	124	52%		

*adjusted for age and year of biopsy. Bold values indicate statistical significance at the p < 0.05 level.

**Table 3:** Distribution of number of lesions (scores) by case/control status, the Detroit BBD Cohort.

	Control		Case		p value
**Score as a continuous predictor**					***<0.001***
Mean (std)	1.2	(1.3)	1.7	(1.4)	
Median (range)	1	(0,6)	1	(0,6)	
**Score as a categorical predictor, N (%)**					***<0.001***
Score 0 (no lesions)	1149	36%	56	24%	
Score 1	971	30%	65	27%	
Score 2	562	17%	49	21%	
Score 3	339	11%	38	16%	
Score 4	146	5%	19	8%	
Score 5	47	1%	9	4%	
Score 6	9	0%	2	1%	

Bold values indicate statistical significance at the p < 0.05 level.

**Table 3** describes the distribution of number of BBD lesions which is reported as both a continuous and categorial variable. The mean score for cases was higher compared to controls (1.7 and 1.2, respectively, p-value <0.001).

**Table 4** depicts the risk of subsequent BC by benign breast lesion score, adjusted for age at biopsy and proliferative or non-proliferative disease. Each additional feature was associated with a 25% higher relative risk of developing BC (RR 1.25, 95% CI 1.10-1.42, p-value 0.001) after adjustment. The BBD lesion score demonstrated its independent predictive role. Additionally, each one-year increase in age at biopsy was associated with a 3% higher relative risk (RR) of developing a BC (RR 1.03, 95% CI 1.02-1.03, p-value <0.001).

**Table 4:** Multivariable logistic regression for the risk of subsequent breast cancer by benign breast lesion score, the Detroit BBD Cohort.

	RR (95% CI)		p value
**BBD lesion Score**	**1.25**	**1.10-1.42**	**0.001**
**Dupont and Page Classification**			0.956
Non-Proliferative Disease	Ref.		
Proliferative Disease without Atypia	1.01	0.71-1.45	
**Biopsy age (per 1 year)**	**1.03**	**1.02-1.04**	**<.001**

Bold values indicate statistical significance at the p < 0.05 level.

**Table 5** provides information on the subsequent BC subtype. Out of the total BC cases, 166 cases (69.7%) were invasive, with hormone receptor positive, HER2 negative cancers the most frequent subtype (55.4%). Triple negative BC, known to be more frequently diagnosed in AA women, comprised 19.0% of invasive cancers in this cohort. For 24 cases (14.5%), subtype could not be determined (insufficient data or tissue available).

**Table 5:** Subtypes of Breast Cancers in the Detroit BBD Cohort.

Subtype	Number (%)
*In Situ*	72 (30.3%)
*Invasive*	166 (69.7%)
HR+, HER2-	92 (64.8%)
HR+, HER2+	15 (10.6%)
HR-, HER2+	8 (5.6%)
TNBC	27 (19.0%)

Note: 24 invasive tumors could not be analyzed, thus the denominator for the subtype analyses is n=142.

## Discussion

In this cohort of nearly 4,000 AA women who had undergone a clinically indicated breast biopsy resulting in a benign diagnosis, we report an increased risk of subsequent BC with several types of benign breast lesions, specifically ductal and lobular hyperplasia, intraductal papilloma, sclerosing adenosis, columnar alterations and radial scars. These observations are similar to those in primarily non-Hispanic white populations, as reviewed by Dyrstad et al. Further, and never examined in a cohort comprised of only AA women, is the finding that as the individual types of BBD lesions found within a single breast biopsy increases, subsequent risk of BC also rises. Multiple types of BBD in a single biopsy are common, with 38% of women included in the National Surgical Adjuvant Breast and Bowel Project’s BC Prevention Trial having more than one type of lesion, similar to the 35.2% reported in our study population (10). Our results are also consistent with those of Worsham and colleagues, who studied the multiplicity of concurrent BBD lesions in another population of women with BBD of similar size from metropolitan Detroit, which was racially diverse (28% were AA). Multiple NPD lesions in a single biopsy were associated with increased risk of BC (RR=1.79, 95% CI: 1.0, 3.21) as were women with multiple PDWA, with a 2.87-fold risk of BC (RR, 2.87; 95% CI, 1.70-4.83) compared to women with only one NPD lesion (11). Importantly, they found that the effects of multiple lesions did not differ by race. More recently, Sherman et al. examined subsequent risk among women who received a percutaneous biopsy with benign findings and considered multiple lesions within the Dupont and Page classification system. They noted a higher risk for both non-proliferative lesions (3 or greater, HR=1.47, 95% CI: 1.14-1.88) as well as proliferative lesions without atypia (3 or greater, HR=2.14, 95% CI: 1.29-3.53) (12). As the majority of breast biopsies are now percutaneous versus surgical excisions, determining whether risk is similar regardless of biopsy type is critical when considering how informative this variable is when modeling BC risk.

At least two BC risk assessment models, the Breast Cancer Risk Assessment Tool (BCRAT, also known as a modified version of the Gail model) and the International Breast Intervention Study model (IBIS, or the Tyrer-Cuzick model) utilize information regarding personal history of benign breast biopsies (13, 14). Only one model has been developed specifically for women with BBD, which extended the BCRAT tool by incorporating detailed pathologic characterization of the benign lesions seen on biopsy, including a number of specific benign lesions as well as overall histologic impression (proliferative vs. non-proliferative) (15). External validation of this model has been limited as most studies do not have access to this detailed pathologic information, thus this model has not been widely utilized. Furthermore, these models have been built and validated in populations of non-Hispanic white women and have lower discriminatory accuracy in other racial and ethnic groups (16, 17). Our findings regarding risk of BC in a group of AA women with BBD highlight the importance of the development of models that provide more concise estimates to inform prevention and screening strategies.

At 30%, the proportion of *in situ* BCs in this population was higher than what is reported in the general population (20% of all BCs) (18). The majority of *in situ* BCs are ductal carcinoma in situ, or DCIS, and the increase in incidence has been attributed to the rise in mammographic screening. As our population of women with BBD have some modality of breast screening, it is not surprising that our proportion of DCIS is higher than in the general population. Among women with invasive BC in this BBD cohort, the majority had ER/PR+, HER2- cancers, whereas 19.0% of invasive tumors were classified as triple negative BC (TNBC). The proportion of TNBC cases in our cohort is slightly lower than what has been reported among non-Hispanic black women nationally (25.6%) (19) and what was reported by Newman et al. in the only other BBD cohort that contains a large proportion of AA women (24.2%) but within the same range (20). While most risk models have grouped together all subtypes of BC, or examined hormone positive cancers, less work has focused on TNBC, a subtype that is more common in AA women regardless of a prior history of BBD.

Our study has notable strengths, as the only cohort focused on benign breast disease and subsequent BC risk in the AA population. Furthermore, all slides (compared to a subset) from the BBD biopsy were re-reviewed by a breast pathologist versus relying on pathology reports. As the risk associated with atypical hyperplasia has already been well-defined and usually requires further treatment or surveillance, we removed this group with the highest risk, allowing for the examination of other types of BBD lesions separate from this established risk, or interventions that may have lowered the risk of subsequent BCs. Additionally, the study encompassed a wide spectrum of BBD lesion types allowing the examination of BBD multiplicity. The cancers that developed were identified through a population-based cancer registry, which allowed access to pathologic details (i.e., receptor status) when tissue was unavailable for testing.

In addition to these strengths, our study also had limitations. As a retrospective cohort study, we did not contact the women included in the BBD cohort. Specifically, we do not have detailed information regarding other risk factors associated with BC, such as family history, BRCA1/BRAC2 status and other reproductive factors included in risk models. Thus, we do not have the ability to develop our own model or test other models in this BBD cohort. We also may have missed some BC cases if the study participant left the tri-county area covered by the cancer registry, although an analysis of Southeastern Michigan Census data from 2000 to 2010 suggested most population movement was within the tri-country area (data not shown). This potential information bias would result in moving the risk estimates towards the null. Additionally, these results, while obtaining cancer data from a population-based registry, are still from a single geographical area. Thus, the results may not be generalizable to other AA women in the United States or women of African ancestry residing elsewhere. Lastly, the methodology employed to formulate the lesion multiplicity assessment (i.e., the benign breast score) assumes uniform risk across all lesions, which is unlikely from a biological perspective.

In conclusion, our study affirms the increased BC risk in AA women with BBD, particularly in those with multiple lesions. There is a clear need to better characterize factors associated with risk in women with BBD. In 2019, the U.S. Preventive Services Task Force recommended the utilization of BC risk assessment models to offer BC chemoprevention in higher risk women (recommendation level B), and specifically highlighted those with BBD: *“…This recommendation applies to asymptomatic women 35 years and older, including women with previous benign breast lesions on biopsy…”* despite the paucity of research in women with BBD as related to the current risk models (21). Inclusion of diverse populations, particularly those which bear a disproportionate burden of disease, are critical to ensuring evidence-based research and prevention approaches benefit all.

## Data availability statement

The raw data supporting the conclusions of this article will be made available by the authors, without undue reservation.

## Ethics statement

The studies involving humans were approved by Wayne State University Institutional Review Board. The studies were conducted in accordance with the local legislation and institutional requirements. The ethics committee/institutional review board waived the requirement of written informed consent for participation from the participants or the participants' legal guardians/next of kin because Retrospective tissue study exemption 4.

## Author contributions

VPat: Writing – original draft. JR: Data curation, Formal analysis, Methodology, Writing – original draft. WC: Formal analysis, Writing – review & editing. JB: Data curation, Investigation, Methodology, Writing – review & editing. EA: Data curation, Investigation, Writing – review & editing. BA: Data curation, Investigation, Writing – review & editing. VPar: Data curation, Investigation, Writing – review & editing. AS: Data curation, Investigation, Methodology, Writing – review & editing. SB: Data curation, Investigation, Writing – review & editing. RA-F: Data curation, Methodology, Supervision, Writing – review & editing. MC: Conceptualization, Data curation, Formal analysis, Funding acquisition, Methodology, Project administration, Supervision, Writing – original draft.
